# School for the Instruction of the Blind

**Published:** 1833

**Authors:** 


### XIV. School for the Instruction of the Blind.

It must gratify every friend of humanity, and raise a feeling of laudable pride in the heart of every citizen of Ohio, to hear of the successful operation of the school for the *Education of the Deaf and Dumb*, at Columbus. The effects already produced in the short space of five years, would do credit to an older community than ours; and are an encouraging earnest, of the many blessings it will confer on those who are born to perpetual silence, in the midst of this noisy world.

Among the various advantages that result from such a humane institution, is this: that it awakens the tenderness of the people at large, and predisposes them to new enterprises of beneficence. Under this conviction, we think it a favorable time, to present to the consideration of our *General Assembly*, of the *Profession*, and *Society at large*, the establishment in Columbus, of a sister *Institution for the Education of the Blind.*

The number of these unfortunate persons, we mean to include those only who are children, or were such when they became blind, is far greater than is generally supposed. For several years, we have professionally, been devoted, in an especial manner, to the treatment of Diseases of the Eye, and every month has presented us with new evidences, of the great number of blind children and youth, within our own State. Many of these were born blind, and might, of course, be cured; but a much larger number became so, in infancy or childhood, from inflammation of the eyes, and their cases are generally irremediable. A large proportion of these are the children of poor parents, and belong to the country, where sore eyes prevail more than in the towns, and are too often subjected to no medical treatment, till incurable blindness is produced. Thenceforth, the child becomes a permanent charge on its parents or friends—often on the township—and passes its life, not only deprived of the enjoyments of sight, and all the benefits of intellectual education, but with the painful consciousness, of being a perpetual burthen on those about it; while, if educated, its happiness would be greatly increased, its moral faculties and affections ameliorated, and its bodily powers made available, to its own support.

The education of the Blind, like that of the Deaf, cannot be conducted at home, even when parents, from their wealth, have the necessary leisure. Specific methods and qualified teachers are indispensable; and if such could be obtained, in sufficient numbers, and all who have blind children had the means of compensation, the progress of the pupil, would be in no degree equal to what it is, when associated in a school, with others, laboring under the same infirmity.

It is then, obviously the duty of every State, to institute and support a school for the education of the Blind; in which not only their intellectual and moral faculties should be cultivated, but they should be taught some mechanical occupation, of a useful kind, the prosecution of which would enable them to earn a livelihood, and raise them from a state of dependence on their friends or the community. Such institutions have existed for some time, in Europe, and have lately been established in Pennsylvania, New York and Massachusetts, with the most beneficial results. As yet, there is not one in the Valley of the Mississippi, where the proportion of *poor* blind children, is greater, perhaps, than in any other part of the Union. Ohio has nearly one third of the population of the Great Valley, and why should she not be the first to institute this noble charity? Having nearly completed, and brought into productive operation, several magnificent public works, her present resources are every way adequate to the humble, but still dearer object, of instructing those unfortunate children, who, without such advantages, can never participate in the enjoyment which every citizen derives from contemplating these honorable achievements.

It is not our design at this time, to go into an analysis of the methods by which the Blind are taught. In point of dignity, and the ingenuity manifested in their invention, they are every way equal to those for the education of the Deaf; and their application calls, equally, for public appropriations and the fostering superintendence of the State.

To all our readers, whether physicians or students of medicine, we would say, do not consider your duties of humanity discharged, by doing professionally what may be in your power, to avert one of the greatest of human afflictions; but as it is an effect of disease, and is therefore connected with the profession, make it your aim to urge upon society, the duty of alleviating the distresses which follow in its train.

To the General Assembly, now deliberating on the public good, we would respectfully address a solicitation still more earnest. They preside over all the interests of the State; some of these are conspicuous, and force themselves upon legislative attention; others are retiring, unobtrusive, and scattered among the habitations of the poor. The intellectual wants of the Blind, are of this class; and for this very reason, must become objects of attention, to a magnanimous legislature. Let the subject, then, of an Institution for the Education of the Blind, be at least referred to a committee, with instructions to collect and report to the next session, the information necessary to legislative action.

